# Protocol of a pilot randomized clinical trial to evaluate nutritional support and rehabilitation on prevention of skeletal muscle mass loss during neoadjuvant chemotherapy in patients with esophageal cancer

**DOI:** 10.1371/journal.pone.0302003

**Published:** 2024-04-18

**Authors:** Yuichiro Miki, Satoshi Nishi, Tatsuro Tamura, Takumi Imai, Mikio Nambara, Tatsunari Fukuoka, Mami Yoshii, Takahiro Toyokawa, Shigeru Lee, Hisako Fujii, Hisako Yoshida, Mitsuhiko Ikebuchi, Kiyoshi Maeda

**Affiliations:** 1 Department of Gastroenterological Surgery, Osaka Metropolitan University Graduate School of Medicine, Osaka, Japan; 2 Department of Surgery, Fuchu Hospital, Osaka, Japan; 3 Department of Medical Statistics, Osaka Metropolitan University Graduate School of Medicine, Osaka, Japan; 4 Department of Surgery, Higashisumiyoshi Morimoto Hospital, Osaka, Japan; 5 Department of Health and Medical Innovation, Osaka Metropolitan University Graduate School of Medicine, Osaka, Japan; 6 Department of Orthopaedics Surgery, Osaka Metropolitan University Graduate School of Medicine, Osaka, Japan; Fujita Health University: Fujita Ika Daigaku, JAPAN

## Abstract

**Background:**

Subtotal esophagectomy with lymph node dissection followed by neoadjuvant chemotherapy (NAC) is the standard treatment for stage II–III esophageal cancer. Esophagectomy is still associated with high morbidity rates, and reducing these rates remains challenging. Among several complications, postoperative pneumonia (PP) is sometimes fatal, which has been reportedly caused by sarcopenia. Thus, nutritional support and rehabilitation may be promising for preventing skeletal muscle mass loss and reduce the incidence of PP.

**Methods:**

This single-center, randomized, open-label, pilot trial will randomize a total of 40 patients with esophageal cancer in a 1:1 ratio either to ISOCAL Clear + rehabilitation arm or only rehabilitation arm. Although all patients will be educated about rehabilitation by a specialized physician and will be asked to undergo the prespecified rehabilitation program, patients treated with ISOCAL Clear + rehabilitation arm will be supplemented by 400 mL of ISOCAL Clear (Nestlé Japan Ltd, Tokyo, Japan) per day during two courses of NAC with docetaxel, cisplatin, and fluorouracil. Body composition will be assessed using Inbody (Inbody Co., Ltd., Tokyo, Japan) just before starting NAC and surgery. The primary endpoint is the change of skeletal muscle index (SMI) during NAC. Secondary endpoints include (i) body weight, total skeletal muscle mass, appendicular skeletal muscle mass, and lean body mass index changes; (ii) the percentage of ISOCAL Clear continuation; (iii) appetite evaluation; (iv) the percentage of targeted calorie achievement; (v) adverse events of NAC; (vi) postoperative complication rates; and (vii) postoperative hospital stay.

**Discussion:**

This prospective trial assesses the efficacy of nutritional support in addition to rehabilitation during NAC for patients with esophageal cancer. The results will be utilized in assessing whether the effects of nutritional support by ISOCAL Clear are promising or not and in planning future larger clinical trials.

## Introduction

Esophageal cancer (EC) is the fifth most common cause of cancer-related deaths for men and the eighth for women [1]. As the Japan Clinical Oncology Group (JCOG) 9907 study confirmed the survival benefits of preoperative chemotherapy, surgery followed by neoadjuvant chemotherapy (NAC) is the standard treatment for stage II–III EC in Japan [2]. Progress in surgical and postoperative management has improved both short- and long-term postoperative outcomes [2, 3]. Despite these advancements, esophagectomy is associated with high morbidity and mortality rates [4, 5]. Among the complications, postoperative pneumonia (PP) is sometimes fatal; thus, the development of novel preventive strategy will be warranted.

We have reported that preoperative sarcopenia based on the EWGSOP criteria is a significant indicator for predicting PP after esophagectomy in patients with EC [6] because the thoracic wall and abdominal muscles are critical for maintaining airway clearance. In addition, it has been shown that the skeletal muscle mass declines during NAC [7–9]. Thus, several approaches should be attempted to increase the skeletal muscle mass.

As for the nutritional support during chemotherapy, the oral elemental nutritional support by Elental (EA pharma, Tokyo, Japan) increased the continuation rate of adjuvant S-1 for patients with gastric cancer [10]. Body weight loss has been known to be prevented by Elental after patients who underwent gastrectomy [11, 12]. These data reinforce that nutritional support during chemotherapy could be important for maintaining the body weight; however, no evidence has demonstrated the effectiveness of combining nutritional and rehabilitation supports for skeletal muscle mass, especially in patients with EC. The ISOCAL Clear (Nestle) is used in this study because of the following two main reasons: (i) ISOCAL Clear contains adequate branched chain amino acids, which are essential for muscle synthesis, and (ii) its flavor is specially designed for appetite loss and fit to patients who undergo chemotherapy. The impact of nutritional support is stronger with exercise; therefore, we planned a prospective open-label randomized study to evaluate the effects of ISOCAL Clear and rehabilitation for preventing skeletal muscle mass loss.

## Methods

### Objective and design

This trial aimed to explore and evaluate the effects of nutritional support using the ISOCAL Clear in addition to rehabilitation for preventing skeletal muscle mass loss during NAC for patients with EC.

This is a single-center, prospective, open-label, randomized pilot study, conducted at Osaka Metropolitan University Hospital, Department of Gastroenterological Surgery. The patient enrollment period is from March 2023 to August 2024.

Clinicopathological findings of EC are documented based on the Japanese Classification of Esophageal Cancer 11th edition. Performance Status (PS) is evaluated using the Eastern Cooperative Oncology Group (ECOG) criteria. Adverse events are documented using the Common Terminology Criteria for Adverse Events version 5.0.

The study protocol was approved by the institutional ethics committee of Osaka Metropolitan University Graduate School of Medicine (approval number: OCU0040) and was registered in the Japan Registry of Clinical Trials (jRCTs051220167, 13, Feb, 2023).

### Endpoints

#### Primary endpoints

The primary endpoint is the change of skeletal muscle index (SMI) during NAC.

#### Secondary endpoints

Secondary endpoints include (i) body weight, total skeletal muscle mass, appendicular skeletal muscle mass, and lean body mass index changes, (ii) the proportion of the ISOCAL Clear continuation, (iii) appetite evaluation, (iv) the proportion of targeted calorie achievement, (v) adverse events by NAC, (vi) postoperative complication rates after subtotal esophagectomy, and (vii) postoperative hospital stay after subtotal esophagectomy.

### Eligibility criteria

To be eligible for the trial, patients must meet the following criteria:

1. Histologically proven squamous cell carcinoma, adenosquamous carcinoma, or basaloid cell carcinoma
2. Stage II–III
3. Aged ≥20 years
4. ECOG PS of 0 or 1
5. Oral intake ability
6. Preserved major organ function
7. Signed written informed consent

### Exclusion criteria

Patients who meet any of the following criteria are not eligible for the study:

1. Simultaneous or metachronous (within 5 years) double cancers, except for intramucosal tumors treatable with local therapy
2. Active infection requiring systemic therapy
3. Fever of >38°C
4. Pregnant or lactating women or within 28 days of postparturition; men who want their partners to get pregnant
5. Psychological disorders hindering the participation to this clinical study
6. Diabetes mellitus uncontrolled with continuous use of insulin or hypoglycemic agents
7. Severe emphysema, interstitial pneumonia, or pulmonary fibrosis based on chest CT
8. Uncontrolled arterial hypertension
9. Drug history of opioids or steroids
10. History of unstable angina pectoris within 3 weeks or myocardial infarction within 6 months before registration
11. Milk (protein) allergy
12. Other reasons considered as ineligible by the principal investigator

### Randomization

A surgeon introduces the study to the patients at the outpatient clinic, and informed consent will be obtained before the start day of NAC. Patients will be randomized before NAC. Randomization is performed by a study statistician using the stratified permuted block method where the strata were considered based on SMI at enrollment (sarcopenia or not), and sex (male, female). The Research Electronic Data Capture (REDCap) platform, a web-based software platform specifically designed to facilitate data capture for research studies、 was used to allocate patients according to the randomization list. After the study enrollment, patients will be centrally randomized in a 1:1 ratio into ISOCAL Clear + rehabilitation arm and only rehabilitation arm on the REDCap system.

### Treatment

If patients are allocated to the ISOCAL Clear + rehabilitation arm, they will be asked to drink two bottles of ISOCAL Clear (200 Kcal/200 mL/bottle) per day during the two courses of NAC (1–28 days/course). Those patients are not asked to drink ISOCAL Clear during the interval time of chemotherapy, or after surgery. ISOCAL Clear contains 1370 mg leucine, 610 mg isoleucine, and 550 mg valin/bottle. Conversely, if patients are allocated to the only rehabilitation arm, ISOCAL Clear will not be administered.

Patients will attend a lecture about preoperative rehabilitation by a specialized physician. In hospitalization, patients will be asked to perform aerobic training by walking or bicycle ergometer, muscle trainings, and respiratory training, including neck and thoracic wall stretching and incentive spirometry. In addition, a physician instructs patients on how to self-train after discharge with pamphlets. This rehabilitation process will be performed in both groups.

The treatment record will be written to the specific sheet by the patients themselves. The amount of ISOCAL Clear intake will be recorded only in ISOCAL Clear+rehablitation group, while the complete rate of the rehabilitation program is recorded in both groups. This sheet is written in Japanese language. They are administered by resident/fellow doctors in the ward, and validated by an attending surgeon. The information about dietery intake is collected only during in hospital stay period by clinical nutritionist.

### Follow-up and outcomes

**Fig 1:**
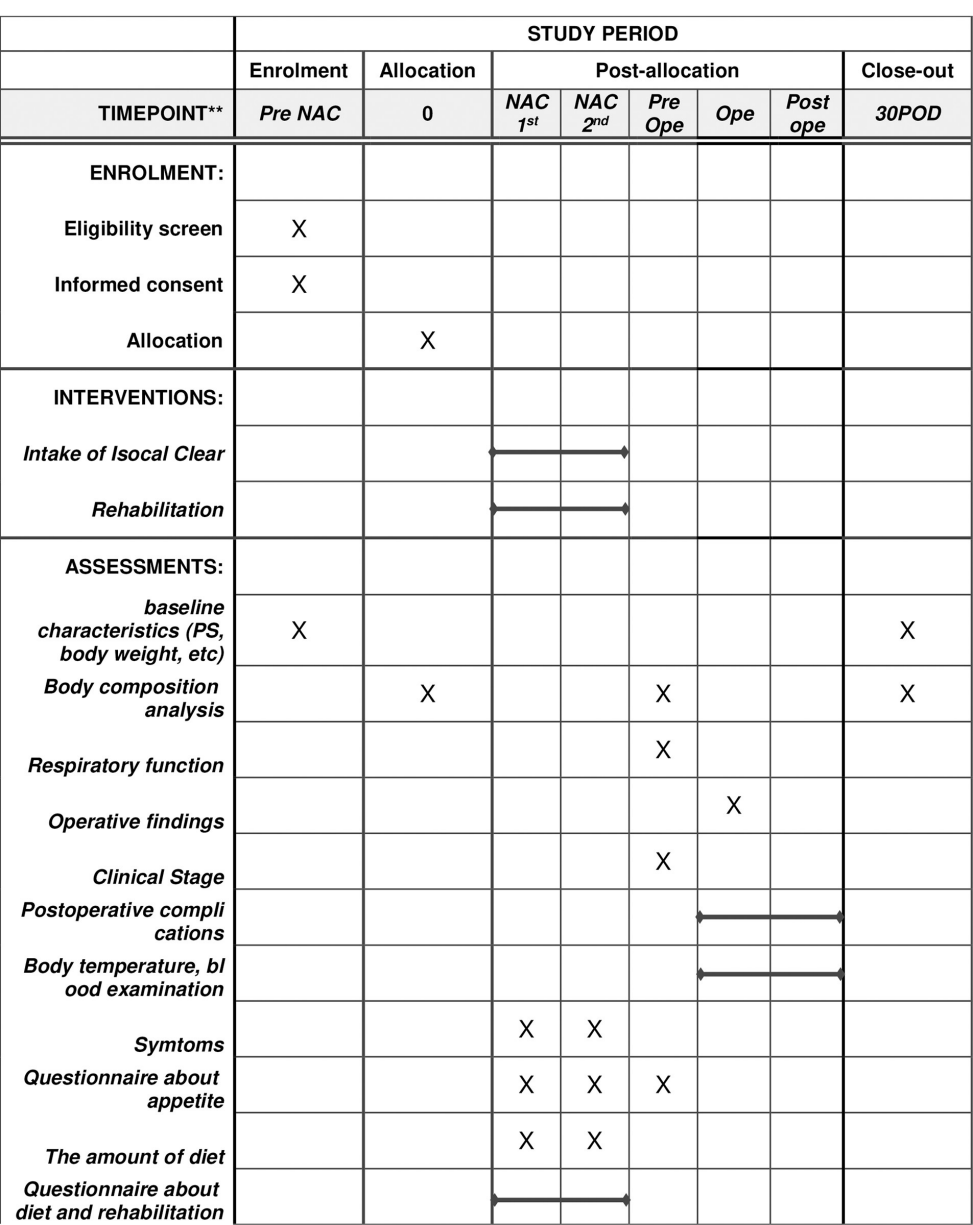
The schedule of enrolment, interventions, and assessments.

Body composition will be assessed using Inbody (Inbody Co., Ltd., Tokyo, Japan) just before starting NAC and surgery. Inbody can assess the total and appendicular skeletal muscle mass and lean fat body mass index. The appetite of patients will be assessed using a FAACT questionnaire [13]. Information regarding the dietary intake will be collected by ward nurses, and a nutritionist will calculate the calories. The assessment schedule during the study is summarized in Fig 1.

### Monitoring

**Fig 2:**
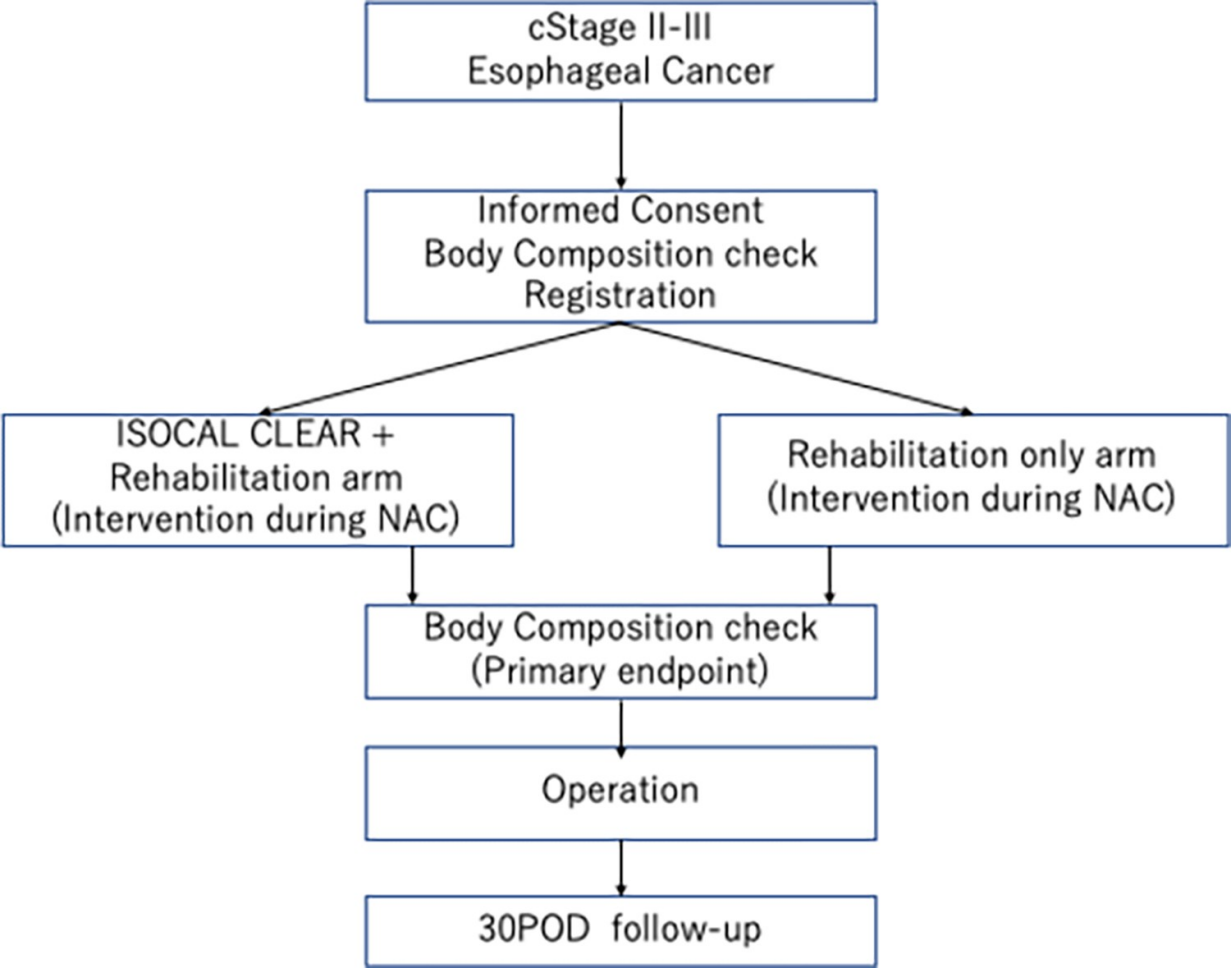
The participant flow diagram.

Central monitoring is planned to be performed using central monitors appointed by the principal investigator. Central monitors review the collected data based on case report forms, and then, reports of aggregate results are generated once a month after enrolling ≥10 participants to ensure that study protocol is followed and data are collected accurately. On-site monitoring will be performed using site monitors appointed by the principal investigator. On-site monitors are mainly responsible for the process of obtaining informed consent and determining eligibility, the management of intervention foods, and the preservation of clinical trial documentation. Total study schema is shown in Fig 2.

### Statistical considerations

As a pilot study, the sample size was set as 40 based on the feasibility of recruitment within 1.5 years. The number of potentially eligible patients in the hospital was approximately 60 per year, and the consent to participate was assumed to be obtained from approximately 60% of them. Assuming the nutritional support by ISOCAL Clear would potentially increase the patients’ SMI by 0.4 kg/m^2^ and standard deviation of SMI change of approximately 0.5 kg/m^2^ based on the experience in the hospital, the sample size 40 was estimated to have a power of 70% to detect increased SMI with alpha level of 0.05.

Statistical analyses will be conducted basically on the intention-to-treat basis. For postoperative endpoints, analyses will be conducted for patients who completed esophagectomy. The amount of ISOCAL Clear intake will be described for the ISOCAL Clear + rehabilitation arm. For all endpoints and changes from baseline at each visit, basic descriptive analyses are performed by the treatment group. The SMI changes during NAC will be analyzed using analysis of covariance (ANCOVA) models with adjustments of baseline values and compared in the treatment groups. Among the secondary endpoints, body weight, total skeletal muscle mass, appendicular skeletal muscle mass, lean body mass index, and appetite score by FAACT will be analyzed similarly. If any substantial deviations from the assumptions of ANCOVA are observed, analysis with more flexible assumptions will be performed. The achievement of targeted calories, adverse events by NAC, postoperative complication, and postoperative hospital stay will be described using basic summary statistics in the treatment groups and analyzed in an exploratory manner.

Subgroup analysis is not planned due to the limited sample size. No statistical correction for multiple comparisons will be made due to the exploratory nature of the study. All statistical analyses will be performed using R software version 4.0 (R Core Team, 2023) or later.

## Discussion

This is a prospective, single-center, open-label, randomized trial for assessing the efficacy of nutritional support besides rehabilitation during NAC for patients with EC. The patient enrollment has started in March 2023, and we have planned to complete the recruitment in August 2024.

While neoadjuvant chemoradiothrapy has been adopted as standard treatment for advanced EC in western countries, NAC followed by surgery has been considered as standard treatment strategy in Japan. The JCOG9907 study compared the preoperative chemotherapy (NAC group) with postoperative chemotherapy (adjuvant group) in patients with stage II–III EC [2]. The 5-year overall survival rate was 55% and 43% in the NAC and adjuvant groups, respectively. As a result, NAC with cisplatin and fluorouracil (5-FU) was regarded as the standard treatment. Since 2012, JCOG started the new trial to confirm the superiority of docetaxel and cisplatin plus 5-FU (DCF regimen) over cisplatin plus 5-FU (FP regimen), and the superiority of cisplatin plus 5-FU with chemoradiotherapy over cisplatin plus 5-FU as preoperative therapy (JCOG1109, NExT trial) [14]. Consequently, the preoperative DCF regimen showed significantly better prognosis than the FP regimen, whereas the FP+RT treatment did not show survival benefits compared with preoperative FP regimen. Thus, preoperative NAC by the DCF regimen is the current standard treatment for stage II–III EC, which was adopted for this trial.

Several studies reported that preoperative sarcopenia is a significant risk factor of PP after esophagectomy [15, 16], and we have reported that EWGSOP criteria and our modified version showed better predictive values than others [6]. In addition to preoperative condition, preNAC sarcopenia is also significantly associated with PP (data in submission). Furthermore, previous studies reported that a slight decrease of skeletal muscle mass was consistently observed during NAC. Accordingly, we considered that the strategy to prevent muscle mass loss during NAC could be meaningful for reducing PP.

Nutrition status is a crucial factor for completing chemotherapy, and it has been reported that malnutrition is associated with chemotherapy-induced adverse events in the treatment for colorectal or gastric cancer [17, 18]. Patients with EC often have difficulties in oral food intake, and nutritional support could be more beneficial during NAC course than the other cancer types. ISOCAL clear is not a drug but a food, and its tolerability has been reported at a medical congress. Its flavor should fit for the patients with appetite loss, but we plan to evaluate the amount of ISOCAL Clear intake during NAC.

Both exercise and nutrition should be critical for maintaining skeletal muscle mass. Minnella et al. has reported that preoperative nutritional support and exercise improve functional capacity, although their intervention term is not limited to NAC period [19]. At first, we planned to compare four groups, which are (i) Nutrition and Exercise group, (ii) Nutrition only group, (iii) Exercise only group, and (iv) Neither of them. However, there could be fewer cases in one treatment arm, when we adopted the four randomized groups. In addition, we focused on the effect of nutritional support beside exercise and decided to compare (i) Nutrition and Exercise group with (ii) Exercise only group.

As the effect of nutritional support and rehabilitation during NAC period has not fully been investigated in the randomized trial setting, our results will offer the novel insight on the EC treatment. By referring to our results, larger clinical trial where complication rate is set as primary endpoint should be planned in future.

## Supporting information

S1 File (DOCX)
