# Supplementary material for: Protocol of a pilot randomized clinical trial to evaluate nutritional support and rehabilitation on prevention of skeletal muscle mass loss during neoadjuvant chemotherapy in patients with esophageal cancer
Source: PLoS One. 2024 Apr 18;19(4):e0302003. doi: 10.1371/journal.pone.0302003 (PMC11025928; doi:10.1371/journal.pone.0302003)
Supplement: S1 File — (DOCX) [file pone.0302003.s001.docx]

**「臨床病期 Stage II/III食道癌症例に対する術前化学療法中アイソカルクリア服用およびリハビリテーションによる骨格筋量低下予防効果に関するランダム化比較試験-探索的研究-」**

**について**

説明文書

| この冊子は、担当医師によるこの臨床研究の内容についての説明を補うものです。  この臨床研究についてわかりやすく説明しますので、内容をお読みいただき、担当医師の説明を聞かれた後、十分に考えてからこの臨床研究に協力するかどうかをご自身の意思によってお決めください。  また、何かわからないことや疑問に思うことがありましたら、どんなことでも結構ですので、遠慮なく、何度でも担当医師にご質問ください。  《　この臨床研究の問い合わせ・相談窓口　》  臨床研究責任医師氏名（所属） 　　前田　清　　（　　　消化器外科　　）  相談窓口医師氏名　　（所属）　 　　西　智史　（　　　消化器外科　　）  相談窓口医師氏名　　（所属）　 　　三木　友一朗　　（　　　消化器外科　　）  担当医師氏名（所属）　　　　　　　　 　　　（　　　消化器外科　　）  　　　　連絡先　06-6645-3835 　　　Fax　06-6645-6450 |
| --- |

この研究は、当施設の認定臨床研究審査委員会で審議され承認され、厚生労働大臣に実施計画を提出した上で実施しています。

認定臨床研究審査委員会は、当施設の病院長により設置され、当施設内外の医学、薬学などの専門家に加え、倫理等の専門家や、当施設とは利害関係のない外部委員からなります。また、認定臨床研究審査委員会は、研究を行う医師や製薬企業から独立し、倫理的、科学的および医学的、薬学的観点から、研究を実施または継続することが適切であるかどうかについて調査審議し、その結果を病院長に報告します。

当施設の認定臨床研究審査委員会に関する資料（認定臨床研究審査委員会の手順書、委員名簿、審査された内容および審査結果など）は閲覧することが可能ですので、ご希望がありましたら遠慮なく担当医師または上記相談窓口医師へお伝えください。

大阪公立大学医学部附属病院 臨床研究審査委員会

住所：〒545-0051 大阪府大阪市阿倍野区旭町 1-2-7

あべのメディックス 6 階

担当部署：大阪公立大学医学部・附属病院運営本部 研究推進課

担当部署電話番号：06-6645-3456

大阪公立大学医学部附属病院 臨床研究審査委員会承認

第1版　2022年 10月26日作成

目次

1) はじめに 3

2) この研究の目的 3

3) この研究の対象となる方 4

4) 研究の方法（研究の流れ） 5

5) 研究の予定期間と参加予定人数 10

6) 個人情報の保護について 10

7) 研究への参加により、予想される利益および不利益 10

8) 試験に参加しない場合の治療について 11

9) 費用について 11

10) 健康被害が発生した場合 11

11) 研究協力の任意性と撤回の自由について 12

12) 研究成果の取り扱いについて 12

13) 本試験に関するについて 12

14) 資金源 13

15) 相談窓口 14

# はじめに

　この説明文書は、食道がんに対する術前化学療法中における経口栄養剤の効果について明らかにする「臨床研究」について説明したものです。以下の説明をよくお読みになって、この臨床研究について十分にご理解いただいたうえで、参加されるかどうかをご判断ください。研究に参加されるかどうかは、あなたの自由です。この臨床研究に参加してもよいと思われた場合には、巻末の「同意書」にご署名いただきますようお願い致します。

　この研究に参加されなくても今後の治療において、不利益を受けることはありません。また、この臨床研究の参加に同意された後でも、治療が開始されてからでも、あなたが希望されれば、いつでもやめることが可能です。

この研究について判らないことがありましたら、担当医師や相談窓口にお気がねなくご相談ください。

# この研究の目的

　本研究では食道がんの術前化学療法の期間中に、経口栄養剤であるアイソカルクリアという製品を摂取することで、手術までの待機期間中に筋肉量の低下を予防できないかを検討することを目的としています。アイソカルクリア^*^は、筋肉の合成に必須である分枝鎖アミノ酸を多く含む市販されている製品です。

あなたは、進行食道がんと診断され、手術による治療（食道切除術）の適応と考えられています。手術に際しては食道および胃の一部の切除および周囲のリンパ節を切除する事が基本となります。また手術の前に抗がん剤治療を行うことが、より良い治療成績につながることが分かっています。

　万全の注意を払って手術を行いますが、食道がんに対する手術では約40%に術後合併症が発生すると言われています。その中でも注意が必要な合併症の一つに術後肺炎があります。術後肺炎は術前の時点での筋肉量が低下した状態（サルコペニアとよびます）と関連があることが知られており、手術の前に筋肉量を落とさない工夫が、術後肺炎の予防の上で重要な可能性があります。一方で、筋肉量の低下を予防する方法ついては未だに十分には分かっていません。

*アイソカルクリアについて

食欲が落ちている方やタンパク質を摂取したいと考えている方に、すっきり飲みやすい飲料（食品）としてネスレ社によって開発されました。1本200mlでタンパク質10g、200Kcalを含んでいます。ピーチ風味とレモンティー風味の2つのフレーバーがあります。

# この研究の対象となる方

1. 食道癌と診断されている方
2. Stage II-IIIと診断されている方
3. 20歳以上の方
4. 十分な活動ができる方
5. 飲水、内服が可能な方
6. 採血結果にて血球数に異常なく、臓器機能に異常ない方
7. 研究参加に対する説明文書による同意が患者さんご自身でできる方

# 研究の方法（研究の流れ）

4-1）試験の流れ（図1）

この試験に参加する事に同意された後、術前化学療法を行います。ご参加頂いた全員に、リハビリテーションのプログラムを行って頂きます。

リハビリテーションに加えて栄養剤の追加投与を行うかどうかは登録の際に「無作為」に決まります。つまり、あなたや担当医が選ぶのではなく、五分五分の確率で治療法が決まります。あなたや担当医が治療法を選ぶと、その意思が影響して比べたい治療法の患者さんの特徴に偏りが生じてしまい、正しい臨床試験の結果を得る事ができません。この方法は、どちらが良いか分かっていない治療法を比べるには最もよい方法と考えられており、世界中の臨床試験で採用されています。

図1　試験のながれ

試験への参加を同意

40人の患者さん


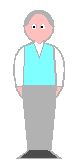

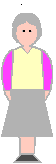

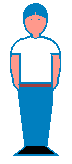

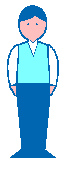

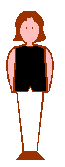

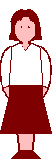

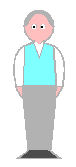

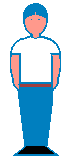

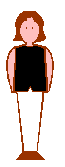

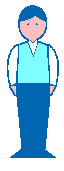

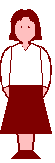

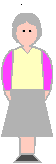


食道癌に対する根治切除前に

体組成に関する評価を行う。

進行食道癌と診断された方

アイソカルクリア服用なし

アイソカルクリア服用あり

20人の患者さん

20人の患者さん

術前化学療法中、リハビリを行う。

体組成の評価を行う。

- 術前化学療法について

Stage II-IIIの食道がんの患者様は、手術の前に化学療法を施行することが、より良い治療成績につながることが分かっています。

　ドセタキセル、シスプラチン、5-FUという薬剤の組み合わせで治療を行う予定です。

　術前化学療法は2コース（1コース：２８日間）行うことが一般的で、その2コースの間に全ての研究への参加される皆様にリハビリテーションを行って頂き、半分の患者様には栄養剤であるアイソカルクリア（ネスレ日本社）を服用して頂きます。

- 手術および術後の一般的な経過

　手術では食道のほとんどと胃の一部を切除し、周囲のリンパ節を定められた範囲において切除を行う郭清を行います。手術にかかる時間は8〜10時間程度です。

　手術自体は全身麻酔で行うため、その間は痛みはありません。麻酔がきれた時には傷が痛む事もありますが、痛み止めによって対処できます。手術後の数日は、点滴で栄養をとります。日が経つにつれ、液体、柔らかいもの、固形物の順に食事をとれるようになります。入院期間は、手術前に2日程度、手術後に2〜3週間程度です。

この臨床試験への参加の有無によって、術前化学療法や手術の内容が変わることはありません。

＜試験への登録後の流れ＞

- 栄養剤摂取なし

　あなたが「栄養剤服用なし」の治療を受けられる場合は術前化学療法中、栄養剤は使用されません。

- 栄養剤摂取あり

　あなたが「栄養剤服用あり」の治療を受けられる場合は術前化学療法の期間中、アイソカルクリアという栄養剤 を1日2回服用して頂きます。

　アイソカルクリアはネスレ日本社から市販されている清涼飲料水で、必須アミノ酸と言われる筋肉合成の材料を多く含んでいる製品です。飲みやすいフレーバーで、常温で保存出来ますが、お飲みになる時は冷やして頂いても結構です。

どちらの群に割り当てられても、術前化学療法中にリハビリテーションを行なって頂きます。

＜リハビリテーションについて＞

リハビリテーションについては、術前化学療法の各コースの開始前に、リハビリテーション科外来を受診して頂きます。その際に、歩行訓練、上下肢、体幹の筋力強化、呼吸筋訓練などについてリハビリテーション担当医から説明があります。

リハビリテーションの詳細については別紙をご参照ください。

その後、術前化学療法期間中はご自宅で、指導内容、別紙を参考にリハビリテーションを行なって頂くこととなります。概ね30-60分/日の運動になります。

＜検査の内容と期間＞

試験への登録前に、Inbodyという体組成測定用の機器で、筋肉量などの評価を行います。また術前化学療法が終了し手術を行う直前および手術後30日頃に、同様に筋肉量などの評価を行い比較を行う予定です。その他にも採血や、臨床病期を確認するための内視鏡検査、CT検査も行われますが、これらはこの研究に参加されない場合も同様です。

　術前化学療法中には食事量や、食欲に関するアンケートにお答え頂く予定となっています。

　手術の後は定期的な採血検査、体温測定などが通常診療の範囲で行われる予定ですが、これらの結果の一部を本研究に利用させて頂きます。

　詳細については以下の表を参考にしてください。

<Inbodyの機器および測定のイメージ図>


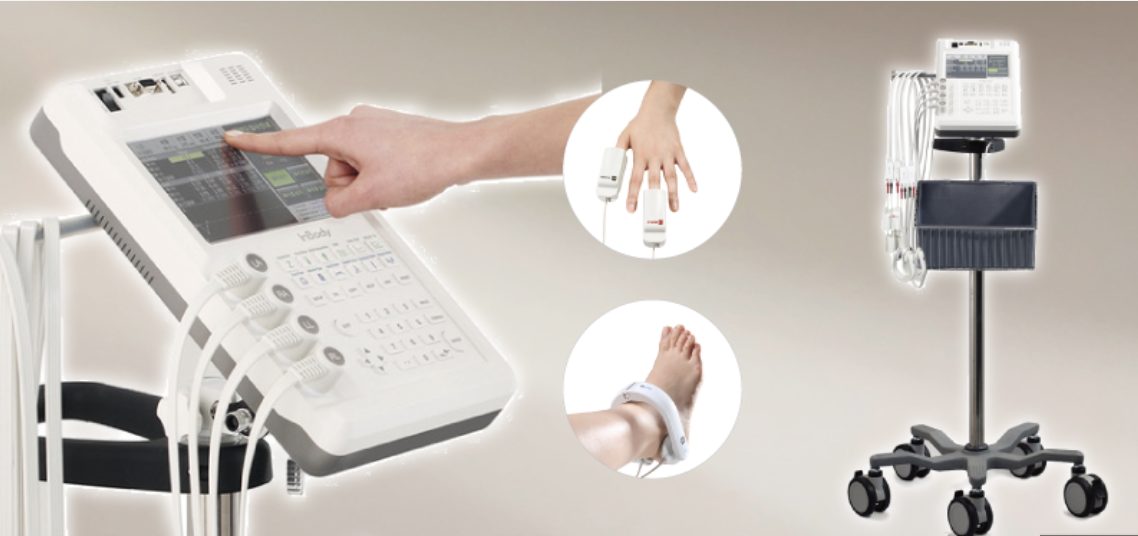


手指および足に電極を装着し測定を行います。

表：検査スケジュール

| 項目/時期 | 登録前 | 登録時 | 術前化学療法前 | 術前化学療法開始日 | 術前化学療法各コースのうち1週間 | 術前化学療法各コース終了時 | 手術前 | 手術日 | 術後　　1日目 | 術後　　2日目 | 術後　　3日目 | 術後　　6日目 | 退院 | 術後30日 |
| --- | --- | --- | --- | --- | --- | --- | --- | --- | --- | --- | --- | --- | --- | --- |
| 体組成評価（骨格筋量） |  | ● |  |  |  |  | ● |  |  |  |  |  |  | ● |
| 呼吸機能検査 |  |  |  |  |  |  | ● |  |  |  |  |  |  |  |
| 体温 |  |  |  |  |  |  |  |  | ● | ● | ● |  |  |  |
| 採血検査 | ● |  | ● |  |  |  |  |  | ● |  | ● | ● |  |  |
| 内視鏡検査、CT検査 |  |  | ● |  |  | ● |  |  |  |  |  |  |  |  |
| アンケート調査 (食欲に関する) |  |  |  | ● | ● |  | ● |  |  |  |  |  |  |  |
| 日誌による記録 |  |  |  | ● | | |  |  |  |  |  |  |  |  |

下線の項目のみが、本研究のために追加する項目であり、その他は通常診療でも行われる検査です。

4-2)本試験に伴う危険性・合併症

　今回の臨床研究で用いる栄養剤は医薬品ではなく、食品であり、予想される副作用はありません。ただし誤って飲食物を気管内に入れてしまうや消化器症状など、間接的な副作用の発生の可能性は否定できません。またリハビリテーションを施行することによる転倒、訓練時の疼痛などは予想される副作用です。

本試験において行う、アイソカルクリアの服用による予想される副作用はありませんが、食道癌に伴う狭窄症状が強い時に摂取することで誤嚥が起こる危険はあります。

リハビリテーションに伴い、転倒による外傷 (骨折を含む)や筋肉増強訓練時の局所の疼痛などの可能性があります。

治療中に行う化学療法および手術は以下のような副作用、合併症が起こり得ます。それらの情報についても収集をさせて頂きます。

⚫︎化学療法に伴うもの

　　抗がん剤による代表的な副作用には以下のようなものがあります。

1. 血球減少
2. 食欲不振
3. 下痢
4. 口内炎
5. 悪心、嘔吐
6. 腎機能障害

⚫︎手術に伴うもの

1. 手術中・手術直後

・術前より貧血がひどい方を除けば、輸血が必要になる程の出血をきたすことは稀です。しかし、手術が終わった時に充分確認してもなお、後で出血を認めることも稀にあります。状況により輸血あるいは再開胸、開腹手術が必要になることがあります。

・手術操作中に他の周囲臓器を損傷することが起こりえます。手術中に迅速な対処は行いますが、程度によっては治癒に時間がかかる場合もあります。手術中にはわからない場合もあり、再手術が必要なこともあります。

1. 手術後

・術後肺炎：食道の切除のために、胸壁の一部を切開してアプローチする関係などで、術後に痰を出しにくくなり、肺炎がおこることがあります。多くは抗菌薬や、痰を吸引する処置でよくなりますが、十分ではない場合は気管切開という処置が必要となることもあります。

・：食道を切除した後に再び食べ物がうまく通る道を作るため、食道と胃をつなぐ（吻合）必要がでてきますが、うまくつながらないことがあります。ほころびが小さい場合には絶食していれば自然にふさがりますが、大きい場合には再手術が必要になることもあります。

・：手術後にはリンパ液、血液などの滲出液が胸や腹のなかにたまります。これらは体に不要なもので、通常は体外に出すためにドレーン（管）を留置しますが、万能ではありません。そして、この滲出液に細菌がつくと膿みを作ります。治療は膿を外にだすことなので、針で刺して出す処置、さらには再手術が必要になることもあります。

・：縫合した傷が自然にくっつくのと同じように、腹部の手術をするとお腹の中で癒着がかならず起こります。変な具合に癒着をすると、腸の通過が悪くなり腸閉塞となります。治療は、絶飲食、鼻から管を入れるなどの処置を行いますが、腸がねじれて再開腹手術が必要になる場合もあります。予防のためにも、術後は早期の歩行を開始することが必要です。

・：手術の傷に細菌がつくことです。膿みを出すために傷を一部開くことがあります。

・：稀ではありますが、手術の影響で発生する下肢の血栓が、肺の血管に飛んでつまる状態を肺塞栓症といいます。いったん起こると致命症になる可能性もありますが、術前からの予測は困難なのが現状です。予防するために、手術中には下肢に弾力性のあるストッキングを履いて頂きますが、術後早期からの足の運動・歩行も予防に重要です。

万全の注意を払って手術を行いますが、実際の手術では上記以外にも予想し得ない合併症が起こることがあります。本研究ではそれらの情報についても収集を行わせて頂きます。

4-3）術後の処置・経過観察

　術前に化学療法を施行している期間中に、指定のリハビリテーションを行い、栄養剤摂取を行う群になった際はアイソカルクリアを服用して頂く以外は従来の術前、術後管理と異なる点はありません。術後特別な合併症がなければ、2-3週間程度で退院していただきます。今回の研究では、術前の骨格筋量の変化や術後合併症の内容、頻度、術後30日の時点での骨格筋量などを調べます。今回の臨床試験に参加することにより追加される検査はなく、その他に体への負担が大きい侵襲的な処置等が追加される事もありません。

# 研究の予定期間と参加予定人数

　研究全体としては、jRCT公開から2025年6月30日までの間で、40人を予定しています。

# 個人情報の保護について

この試験に参加された場合、あなたのイニシャル、生年月日やカルテ番号と共にあなたの診療情報が観察期間終了までデータセンターに定期的に報告されます。この研究で得られた結果は学会報告や論文などに発表されたりしますが、あなたのお名前や住所などの個人を特定できる情報は当院から外部に持ち出したり、公表したりすることはありません。ただし、この研究が正しく実施されたかを確認するために本研究の関係者（モニタリング担当者）や認定臨床研究審査委員会などがあなたの診療録を直接閲覧する可能性があります。この場合においてもあなたの個人に関する情報は厳重に守られます。

この試験への参加を同意される場合、上記の通り、モニタリング担当者などが診療録の閲覧についても同意いただいたことになりますので予め御了承下さい。

# 研究への参加により、予想される利益および不利益

私たちは将来の食道癌の患者さんの術後管理において、より有効でしかも副作用や体に負担が少ない治療法を確立するための情報が、この臨床試験を通じて得られることを期待しています。したがってこの臨床試験に参加する事は、よりよい治療法確立のための社会的貢献になります。

この臨床試験に参加していただく患者さんには、「4-2」に記載されているように、本研究に関連した合併症や副作用が生じる可能性があります。私たちはそれらの可能性を低くするために、この臨床試験を慎重に計画しており、臨床試験中も患者さんの不利益が最小になるよう努力をいたします。しかし、このような不利益が起こる可能性をすべてなくすことはできません。

なお、この臨床試験に参加することによる、ご自身への経済的な利益はありません。

# 試験に参加しない場合の治療について

　今回の臨床試験の対象となる患者さんに対しての標準治療は術前化学療法中の栄養剤服用およびリハビリテーションはなしと考えておりますので、試験に参加しない場合、栄養剤服用およびリハビリテーションは行いませんが、その他通常の診療を受けることが出来ます。

# 費用について

　本試験では、参加した皆さまに、術前化学療法中にリハビリテーションを行って頂きます。そのため、リハビリテーション科受診の費用は保険診療の範囲で負担して頂くことになります (3割負担で1500円程度, 2回の予定です)。

　用いられる栄養剤は市販されているもので、食品に分類されるものです。また摂取群に割り当てられた方はこの栄養剤はこちらから提供致しますので、栄養剤の購入に関する患者様の追加の費用負担は発生しません。

# 健康被害が発生した場合

この臨床研究は細心の注意をもって行われますが、試験に関わる治療 (アイソカルクリアの服用およびリハビリテーション)による予測できなかった重い副作用など、万が一健康被害が生じた場合に備えて、補償制度を用意しています。

この臨床試験に参加したことによって、通常の治療では発生しない健康被害にあったと感じられた場合は、担当医師に遠慮なくお伝えください。この臨床試験に参加したことにより生じた健康被害と判断された場合には、補償のお支払いをします。

補償の内容は補償金（死亡補償金および後遺障害補償金）、医療費および医療手当です。

1. 補償金
2. 死亡補償金

死亡された場合には、定められた給付額をあなたの法定相続人の方にお支払いします。

1. 後遺障害補償金

後遺障害が発生した場合には、内容に応じて定められた給付額をあなたにお支払いします。

1. 医療費

この研究に参加したことによる健康被害の治療に要した治療費のうち、健康保険等からの給付を除くあなたの自己負担額を、支払い限度額の範囲でお支払いします。

1. 医療手当

この臨床研究に参加したことによる健康被害の場合、医療費意外の諸手当として医薬品副作用被害救済制度に準じた金額を支払い限度額の範囲でお支払いします。

# 研究協力の任意性と撤回の自由について

この研究の目的にご賛同いただき、研究に協力するか否かは、あなたの自由です。本研究に参加されない場合でも、不利益を被ることはなく、従来通りの治療を受ける事が出来ます。その場合、術前化学療法中は栄養剤服用なしとなります。

たとえ研究に同意されなかった場合でも、その後の治療を受ける上で不利益を受けることはありません。また、いったん同意されたあとでも、いつでもやめることができます。その場合も、その後の治療を受ける上で不利益をうけることはありません。

# 研究成果の取り扱いについて

この研究の成果は、個人情報が明らかにならないようにした上で、学会発表や学術雑誌等で公に発表されることがあります。またjRCTにも結果は公開されます。個々の患者さんに結果をお知らせすることはありませんが、あなたが希望される場合は担当医師に申し出ていただければ、分かり易い形でご説明させていただきます。

# 本試験に関する資金源、について

　利益相反とは、外部との経済的な利益関係等によって、公的研究で必要とされる公正かつ適正な判断が損なわれる、又は損なわれるのではないかと第三者から懸念が表明されかねない事態を指します。

本研究で使用するアイソカルクリアの製造販売業者であるネスレ日本株式会社から研究資金及び物品の提供を受けて実施していますが、認定臨床研究審査委員会で審査・承認を受けた利益相反管理基準及び利益相反管理計画に基づいて適切に管理・公表することとなっています。

　また本研究に参加する研究責任医師、研究分担医師等にネスレ日本株式会社およびリハビリテーションで用いるトリフローの製造販売元である株式会社フィリップスとも個人的関与はございません。今後、個人的関与が発生した際は、速やかに利益相反管理計画を変更し、認定臨床研究審査委員会の審査、承認を受ける予定です。

# 相談窓口

この研究についてもっと詳しく知りたいとき、同意をとりやめたいときや、なにかわからないことがありましたら、いつでも遠慮なく下記の担当医師または相談窓口にご相談下さい。

当院での相談窓口

大阪公立大学医学部附属病院　消化器外科

住所：大阪府大阪市阿倍野区旭町1-4-3

電話：06-6645-3835

研究責任医師：消化器外科　教授　前田　清

研究担当医師：消化器外科　後期研究医　西　智史

研究担当医師：消化器外科　病院講師　三木　友一朗

担当医師 医師名：

同　意　書

医療機関名　　大阪公立大学医学部附属病院　病院長　殿

臨床研究名：「臨床病期Stage II/III食道癌症例に対する術前化学療法中アイソカルクリア服用およびリハビリテーションによる骨格筋量低下予防効果に関するランダム化比較第Ⅱ相試験」

説明内容：

- この研究の目的
- この研究の対象となる方
- 研究の方法（研究の流れ）
- 研究の予定期間と参加予定人数
- 個人情報の保護について
- 研究への参加により、予想される利益および不利益
- 試験に参加しない場合の治療について
- 費用について
- 健康被害が発生した場合
- 研究協力の任意性と撤回の自由について
- 研究成果の取り扱いについて
- 本試験に関する利益相反について
- 資金源について
- 相談窓口

上記の試験について、私が説明しました。

説明担当医署名：

説明年月日： 　令和　　　　　年　　　　　　月　　　　　　日

上記の臨床試験について、担当医師から説明を受けよく理解しましたので、試験に参加します。

患者本人署名：

同意年月日： 令和　　　　　年　　　　　月　　　　　日

同意撤回書

医療機関名　　大阪公立大学医学部附属病院　病院長　殿

臨床研究名：「臨床病期Stage II/III食道癌症例に対する術前化学療法中アイソカルクリア服用およびリハビリテーションによる骨格筋量低下予防効果に関するランダム化比較第Ⅱ相試験」

上記の試験についての同意を撤回します。

患者本人署名：

同意撤回年月日： 令和　　　　　年　　　　　月　　　　　日
